# Adenoid Growths of the Nasopharynx: When and How to Operate

**Published:** 1904-04-30

**Authors:** 


### ADENOID GROWTHS OF THE NASO-PHARYNX: WHEN AND HOW TO OPERATE.

In a paper read before the West London Medico-Chirurgical Society, Mr. Garry Simpson[1] spoke of the large amount of attention the nasopharynx had received during recent years. An incalculable number of individuals at the present time are useful members of society who, without the removal of their adenoids, would have been permanently deaf, besides suffering great inconvenience and discomfort. The growths are found on the posterior, superior, and lateral walls of the nasopharynx, most frequently on the upper curved part of the posterior wall. Ninety per cent. of children with hypertrophied tonsils have adenoids as well. In infants operative measures are required when the child is unable to suck without losing its breath, and when it makes a great deal of noise in its breathing. Having excluded congenital post-nasal occlusion and acute and specific rhinitis, the adenoids should be removed with the smallest curette. In older children repeated attacks of catarrh of the Eustachian tubes and chronic otitis media associated with adenoids are definite indications for operative interference. When a child is obviously suffering in health both mentally and physically, is a habitual mouth-breather, and as a consequence suffers from chronic nasal catarrh, it is imperative that the growths should be removed as early as possible. Youths and adults in which the adenoids have already injured the hearing apparatus permanently are frequently benefited by operation, followed by inflation and massage of the drum membrane. As guides when to operate, the duration, persistency and urgency of the symptoms are of greater importance than the amount of adenoid tissue. If the symptoms are such as not to demand operation a nasal douche should be ordered and instructions given for nasal breathing exercises. Before operation a purge must be given to lessen hæmorrhage, and disinfectant mouth washes may be employed. The patient's general health must be as good as possible. All instruments should be sterilised, and the operation is preferably performed early in the morning, the teeth being carefully examined, as the gag occasionally removes a loose one. The best anæsthetic for a patient over 10 years of age is gas. Somnoform is good, but the cough reflex is rapidly lost. For young children chloroform seems the best, provided it is not pushed to the point of abolishing the cough reflex. The patient is placed in a chair with a head-rest. A Doyen's gag and a laryngoscopic mirror are required. The tonsils are excised first, then the fossæ of Rosenmüller are cleared out with the finger nail, and the growth on the posterior wall removed with a Delstanche curette. An ordinary Gottstein curette being used also if necessary. The head is then pushed well forward and the blood flows into a basin held by a nurse.

Another method is to place the patient on his side with the head resting on a pillow and the face projecting over the edge of the table. The index finger of the left hand guides the largest curette which will pass between the Eustachian prominences into the nasopharynx and up to the septum. A few sweeps are made downwards, and the remaining fragments cleared out with Bronner's curette and the sterilised finger-nail. After the operation the patient should be kept in bed for 24 hours on cold fluids.

Light food should be ordered for a few days, and exposure to cold winds avoided. No douche or spray should be prescribed.

[1] West London Med. Jour., April.

